# Simulation of Spatial and Temporal Patterns of Suitable Wintering Habitat for Hooded Crane (*Grus monacha*) Under Climate and Land Use Change Scenarios

**DOI:** 10.3390/ani15010006

**Published:** 2024-12-24

**Authors:** Zeng Jiang, Mingqin Shao, Jianying Wang

**Affiliations:** College of Life Science, Jiangxi Normal University, Nanchang 330022, China; jiangzeng@jxnu.edu.cn (Z.J.); jianying@jxnu.edu.cn (J.W.)

**Keywords:** wintering period, MaxEnt, habitat suitability, climate change, centroid

## Abstract

In the context of global warming and intensified human activities, the loss and fragmentation of species habitats have been exacerbated. In order to clarify the trends in the current and future suitable wintering areas for hooded cranes (*Grus monacha*), the MaxEnt model was applied to predict the distribution patterns and trends of hooded cranes based on 94 occurrence records and 23 environmental variables during the wintering periods from 2015 to 2024. The results indicated the following. (1) The elevation (Elev, 43.7%), distance to major water (DW, 39.5%), minimum temperature of the coldest month (Bio6, 9.7%), and precipitation of the wettest month (Bio13, 2.6%) were dominant factors influencing the wintering distribution of hooded cranes. (2) Under current climate and land use scenarios, highly suitable areas for hooded cranes in China cover approximately 1.274 × 10^5^ km^2^, primarily located in inland lakes such as Dongting Lake, Liangzi Lake, Poyang Lake, Shengjin Lake, and Caizi Lake in the middle and lower reaches of the Yangtze River, as well as in coastal wetlands such as Chongming East Beach, Shandong Peninsula, Bohai Bay, and Liaodong Peninsula. (3) Under future climate and land use scenarios, the suitable habitat areas (high and moderate suitability) for hooded cranes are projected to contract substantially in the middle and lower reaches of the Yangtze River and expand slightly in the areas of Shandong Peninsula, Bohai Bay, and Liaodong Peninsula. Under the SSP126 (low emissions), SSP245 (medium emissions), and SSP585 (high emissions) scenarios, the average area reduction percentages were 29.1%, 28.8%, and 31.6%, respectively. (4) The increases in Bio6 and water areas in northern China were the main reasons for the shift of the wintering distribution centroid for hooded cranes toward northeastern China. The minor expansion of suitable habitat in the north covers mainly cultivated land, and this singular foraging habitat could intensify both intraspecific and interspecific competition among waterbirds, thus exacerbating the survival risks for hooded cranes. To more effectively protect the wintering population of hooded cranes in China, the restoration of natural habitats and population monitoring in the middle and lower reaches of the Yangtze River should be strengthened. Additionally, nature reserves or protected areas should be established in the northern expansion regions.

## Simple Summary

The MaxEnt model was applied to predict the distribution patterns and trends of hooded cranes (*Grus monacha*) based on 94 occurrence records and 28 environmental variables during the wintering periods from 2015 to 2024. The results indicated that the elevation, water environment (distance to major water, precipitation in the wettest month), and temperature (minimum temperature of the coldest month) were significant factors in the wintering distribution of hooded cranes. Under current climate and land use scenarios, highly suitable areas for hooded cranes in China cover approximately 1.274 × 10^5^ km^2^, primarily in the inland lakes of the middle and lower reaches of the Yangtze River, Chongming East Beach, and coastal wetlands in northern China. Under future climate and land use scenarios, the suitable habitat areas (high and moderate suitability) for hooded cranes are projected to shrink significantly in the middle and lower reaches of the Yangtze River and expand slightly in northern China. The wintering distribution centroid of hooded cranes is also predicted to shift toward northeastern China. Natural habitat restoration and population monitoring should be strengthened to more effectively protect the hooded crane population.

## 1. Introduction

Climate change and human activities have profoundly impacted biodiversity and ecosystem sustainability [1,2,3,4]. The current global surface temperature has increased by at least 1.5 °C compared to temperatures in the mid-20th century [5,6]. Many species have migrated to higher latitudes or altitudes, potentially altering global distribution patterns significantly [7,8,9,10]. Additionally, increased human activities have led to dramatic changes in land use, which can lead to habitat loss and fragmentation for many species [11,12,13,14,15]. Studies have shown that more than 85% of vulnerable (VU) or endangered (EN) mammals, birds, and amphibians in terrestrial ecosystems are impacted by habitat destruction and degradation [16,17]. Interactions between climate and land use changes could further elevate species’ extinction risks [18,19]. Therefore, understanding species’ distribution dynamics under current and future climate and land use scenarios offers critical insights for conservation efforts and guides strategy development and implementation.

Species distribution models (SDMs) use statistical and ecological theories to predict the current and future distribution patterns of species by analyzing the relationships between known distribution points and environmental variables [20]. Commonly used models include the genetic algorithm for rule-set prediction (GARP), the generalized linear model (GLM), and the maximum entropy model (MaxEnt) [20]. Among these, the MaxEnt model requires fewer samples and provides high accuracy when handling complex, multidimensional environmental data. It has been widely used in recent years for species distribution simulation, extinction risk assessment, and protected area planning [6,21,22].

The hooded crane (*Grus monacha*) is listed as a vulnerable species by the International Union for the Conservation of Nature (IUCN) and classified as a first-class national protected species in China [23,24]. The breeding range of hooded cranes is primarily in the Vilyuy and Ussuri river basins in Siberia, Russia [25], while its primary wintering sites in China include Poyang Lake, Shengjin Lake, Caizi Lake, Dongting Lake, and Chongming Dongtan [26]. Hooded cranes, along with other crane species, serve as a crucial indicator of the health of wetland ecosystems within lake environments. These species are highly dependent on wetlands, which provide essential wintering and foraging habitats [27,28]. However, climate change and intensified human activities, such as urban and agricultural expansion, have led to significant reductions in wetland areas and losses of suitable wintering habitats for waterbirds [29,30,31]. The food resources and population size of hooded cranes have declined significantly, with foraging habitats gradually shifting from natural wetlands to paddy fields [32,33]. Studies on the wintering ecology of hooded cranes have mainly focused on population size [33], behavior [24], habitat suitability and foraging ecology [24,26,34,35,36,37,38,39] and gut microbiota [40,41]. However, uncertainties regarding suitable wintering areas and dominant factors influencing hooded cranes under varying climate and land use scenarios hinder effective conservation efforts.

We expect that under future climate and land use change scenarios, the suitable wintering habitats for the hooded crane will contract in southern regions and expand in northern regions, with the distribution centroid shifting northward. This study aims to test this hypothesis by achieving three main objectives: (1) identifying the key factors influencing the distribution of the hooded crane in China, (2) analyzing the spatiotemporal patterns of suitable wintering habitats under current and future climate and land use scenarios, and (3) assessing future trends in habitat suitability and proposing targeted conservation strategies. The results will provide a scientific basis for long-term conservation planning for the hooded crane and its wintering habitats in China.

## 2. Materials and Methods

### 2.1. Occurrence Data Collection

**Figure 1:**
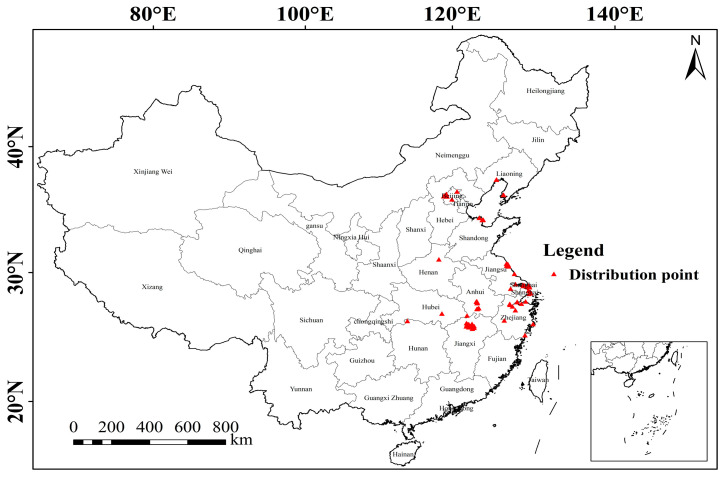
Wintering distribution map of hooded cranes (note: hooded crane occurrence data were sourced from the Global Biodiversity Information Facility (GBIF), China Birdwatching Records Center, eBird, and field surveys (2015–present)).

The occurrence data for hooded cranes in this study were obtained from the Global Biodiversity Information Platform [42], the China Birdwatching Records Center [43], eBird [44], and our field surveys from 2015 to the present. The occurrence records were loaded into ArcGIS 10.8 and overlaid with environmental variables; records from outside the environmental variable layer were removed. To avoid spatial autocorrelation, redundant records within a 1 × 1 km grid were excluded using buffer analysis [45]. Finally, a total of 94 occurrence records remained for analysis (Figure 1).

### 2.2. Species Distribution Range

The species primarily occurs in the middle and lower Yangtze River Plain and the Bohai Sea coastal belt [33,36,42,43,44]. The Yangtze River basin includes the Jianghan Plain in Hubei, the Poyang Lake Plain in Jiangxi, and the Yangtze Delta region, which spans Jiangsu, Zhejiang, and Shanghai. This area experiences a subtropical monsoon climate, characterized by abundant rainfall, an average annual temperature of 14–18 °C, and annual precipitation of approximately 1300 mm [46]. This region serves as a key wintering site for waterbirds along the East Asia–Australasia migratory flyway [46]. Habitats in this region include rivers, lakes, swamps, coastal mudflats, and artificial wetlands, such as reservoirs and ponds [26]. The Bohai Sea coastal belt extends from Weifang in Shandong to Jinzhou in Liaoning, spanning 14 coastal cities across Liaoning, Hebei, Tianjin, and Shandong. This “C”-shaped region has a temperate to warm–temperate climate with distinct seasons: cold winters, hot and rainy summers, an average annual temperature of 8–12 °C, and annual precipitation ranging from 400 to 1000 mm [47]. Important migratory habitats in this area include intertidal wetlands, salt marshes, estuarine wetlands, lake wetlands, and wet meadows [47].

### 2.3. Study Species

The global population of hooded cranes is estimated at 11,600 individuals, with the majority wintering in Izumi City, Japan [26]. The remainder overwinter in the middle and lower Yangtze River region of China and on the Korean Peninsula [23]. During winter, hooded cranes primarily feed on plant materials, including crops and bitter grass (*Vallisneria natans*), while supplementing their diet with mollusks and insects [31]. Due to the conversion of lakes into agricultural land and the transformation of paddy fields into cotton and rapeseed cultivation, the populations of hooded cranes in Dongting Lake and Longgan Lake have nearly disappeared [26]. Additional threats include coastal water pollution and the invasion of intertidal smooth cordgrass (*Sporobolus alterniflorus*), among others [26].

### 2.4. Environmental Data

This study selected 28 variables, including 19 bioclimatic variables, 1 land use variables, elevation, 2 anthropogenic disturbance variables, 2 topographic variables, and 3 variables related to food resources (Table 1). The bioclimatic data comprised 19 climate variables from the World Climate Database [48]. The timeframe for the current climate data spans 1970 to 2000, while the future climate data were sourced from the Coupled Model Intercomparison Project Phase 6 (CMIP6) via WorldClim. CMIP6 represents a significant project in global climate change research, aimed at enhancing understanding of the climate system through comparative outputs of multiple climate models [49]. CMIP6 includes various shared socioeconomic pathways (SSPs) that represent diverse socioeconomic development and greenhouse gas (GHG) emissions scenarios, simulating potential future climate changes [49,50]. For this study, three emission scenarios—SSP126 (low emissions), SSP245 (medium emissions), and SSP585 (high emissions)—were selected for the periods 2040–2060 (2050s) and 2060–2080 (2070s). The HadGEM3-GC31-LL climate system model, developed by the UK Met Office, was chosen for its broad application in climate scenario prediction and analysis [20,51].

Land use data were obtained from the “Figshare” platform [52], simulating 1 km resolution land use patterns across various carbon emission scenarios from 2020 to 2100, enabling the capture of global land use changes under combined climate and socioeconomic influences [53]. The 2020 land use data were selected as the baseline variables, while data for the 2050s and 2070s under the SSP126, SSP245, and SSP585 scenarios were regarded as the future land use variables. Euclidean distance calculations were applied to water bodies and towns to derive proximity measurements. Elevation data (ELE) were derived from the digital elevation model (DEM) available on the Geospatial Data Cloud Platform [54].

To mitigate potential multicollinearity among the climate variables, the 19 variables were initially modeled to determine the contribution rates, then “sampled” in ArcGIS according to the distribution location, and analyzed through correlation analysis. Variables with a Spearman’s correlation coefficient *r* ≥ |0.8| and low contribution rates were excluded [55]. Finally, all the variables were exported as model-supported ASCII files, standardized to the WGS_1984 coordinate system, at a resolution of 30″ (~1 km) and with a consistent boundary mask.

### 2.5. MaxEnt Model Construction and Evaluation

The occurrence records and environmental data were imported into MaxEnt 3.4.1 for analysis. For modeling, 75% of the occurrence records were randomly selected, with the remainder reserved for model verification. The response curves and jackknife method were employed to assess the contribution rates of environmental variables. The modeling process was repeated 10 times, with the average of these iterations used as the final result [55,56]. Response curves illustrate the relationship between the species presence probability and environmental variables, helping to clarify how species respond to varying environmental conditions. The jackknife method identifies the most critical environmental factors for predicting species distributions by iteratively excluding each environmental variable and observing changes in the model performance to determine each variable’s importance [20].

The model accuracy was assessed using the receiver operating characteristic (ROC) curve and the area under the ROC curve (AUC). The AUC values range from 0 to 1: failure (AUC ≤ 0.6), poor (0.6 < AUC ≤ 0.7), fair (0.7 < AUC ≤ 0.8), good (0.8 < AUC ≤ 0.9), and excellent (0.9 ≤ AUC < 1) [51,57].

### 2.6. Dominant Factors and Suitable Areas

The MaxEnt model calculates the contribution rate for each environmental factor, indicating its relative importance in predicting the species distribution. Environmental factors with higher contribution rates are more informative in explaining the species distribution [20,49]. Additionally, the replacement importance assesses the influence of each factor by randomizing the environmental variables and observing the model performance changes [56]. The importance of environmental variables to the distribution of hooded cranes was assessed using the contribution rates and the results of the jackknife test [56].

Additionally, response curves help identify habitat suitability intervals, with areas having a probability of presence greater than 0.5 generally considered suitable [20]. The model outputs a suitability index layer with values ranging from 0 to 1, where higher values indicate greater habitat suitability [55]. The habitat suitability levels were classified into four categories—high, moderate, poor, and unsuitable areas—using natural breaks [58].

### 2.7. Changes in the Suitable Areas and Centroid Shift

The “SDM” toolkit in ArcGIS 10.4.1 was used to compare the distribution range of suitable areas for hooded cranes under current and future climate scenarios, categorizing them into stable, expanding, and contracting areas. The suitability layer was converted into binary data using the moderate and high suitability values as thresholds, and the “Centroid Changes” tool calculated the centroid for the suitable habitat distribution across different time periods, along with the migration distances between these centers and the current centers [20].

## 3. Results

### 3.1. Model Accuracy

**Figure 2:**
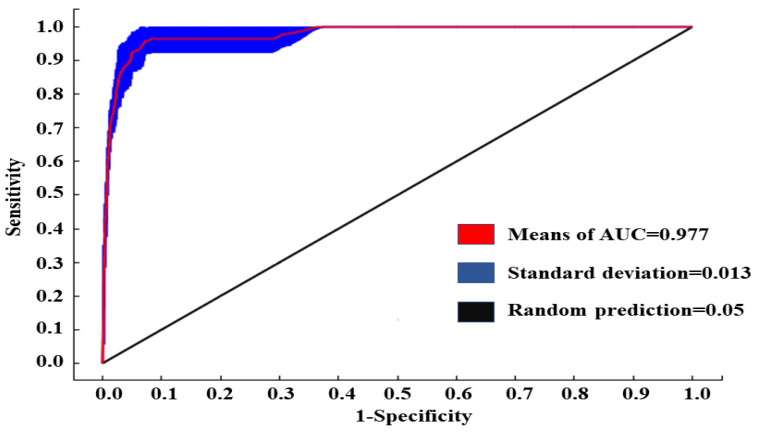
The ROC curve predicting the overwintering distribution of hooded cranes based on the MaxEnt model (note: the ROC curve (receiver operating characteristic curve) assesses the model’s performance by plotting the relationship between 1—specificity (false positive rate) on the x-axis and sensitivity (true positive rate) on the y-axis; the AUC (area under the curve) represents the overall accuracy of the model, with values closer to 1 indicating better performance).

The AUC value of this model was 0.977 and the standard deviation was 0.013, indicating high prediction accuracy, providing excellent and reliable prediction results for the suitable habitats for hooded cranes in the current and future winters (Figure 2).

### 3.2. Importance of Environmental Variables

Following the initial screening, a total of seven environmental variables were retained for modeling suitable wintering areas for hooded cranes (Table 2). The results from 10 MaxEnt simulations indicated that the elevation (Elev, 43.7%), distance to major water (DW, 39.5%), minimum temperature of the coldest month (Bio6, 9.7%), and precipitation of the wettest month (Bio13, 2.6%) contributed more than 2% each, with a combined contribution of 95.5% for these four environmental factors.

**Figure A1:**
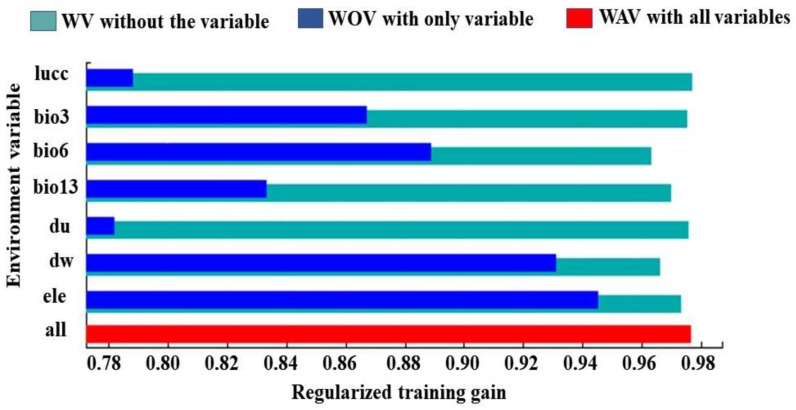
The jackknife test results of the environmental variables affecting the wintering distribution of hooded cranes.

The jackknife method results (Appendix A Figure A1) indicated that the elevation (Elev, 0.945) yielded the highest formalized training benefit when used alone in the model, followed by the distance to major water (DW, 0.932), minimum temperature of the coldest month (Bio6, 0.888), isothermality (Bio3, 0.867), and precipitation of the wettest month (Bio13, 0.833). Removing the minimum temperature of the coldest month (Bio6, 0.966) resulted in the largest drop in the gain values, followed by the distance to major water (DW, 0.968), precipitation of the wettest month (Bio13, 0.97), elevation (Elev, 0.973), isothermality (Bio3, 0.975), slope (du, 0.976), and land use classification (LUCC, 0.976). Overall, the elevation (Elev), distance to major water (DW), minimum temperature of the coldest month (Bio6), and precipitation of the wettest month (Bio13) were identified as the key factors shaping the wintering distribution of hooded cranes.

### 3.3. Response Curves of Environmental Variables

**Figure 3:**
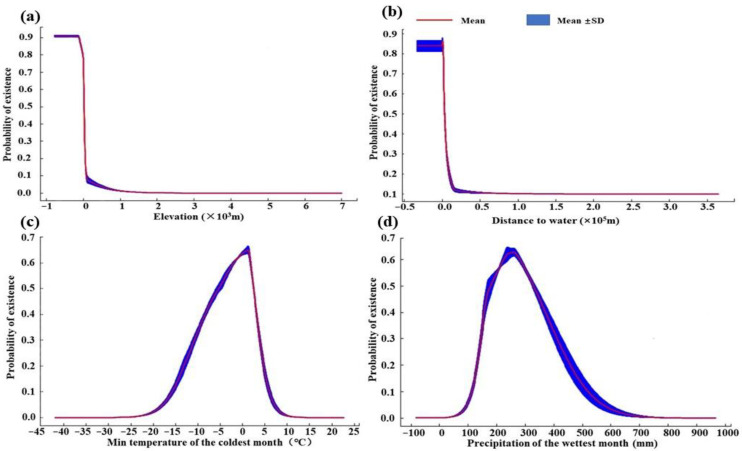
Response curve of hooded cranes to dominant environmental variables (results are based on the logistic output of the MaxEnt model for the hooded crane: (**a**) elevation, (**b**) distance to water, (**c**) min temperature of the coldest month and (**d**) precipitation of the wettest month).

Hooded cranes preferred overwintering at elevations below 22.3 m, as the probability of presence declined sharply above this threshold (Figure 3a). The presence probability remained above 0.5 when the distance to major water bodies was less than 2.26 × 10^3^ m, decreasing sharply as the distance increased (Figure 3b). The probability of presence was above 0.5 when the minimum temperatures in the coldest month ranged from −5.3 to 2.4 °C (Figure 3c). Additionally, the cranes favored areas with 178.2–328.5 mm of precipitation during the wettest month, with the presence probability decreasing significantly when the precipitation levels moved outside this range (Figure 3d).

### 3.4. Current Suitable Wintering Areas

**Figure 4:**
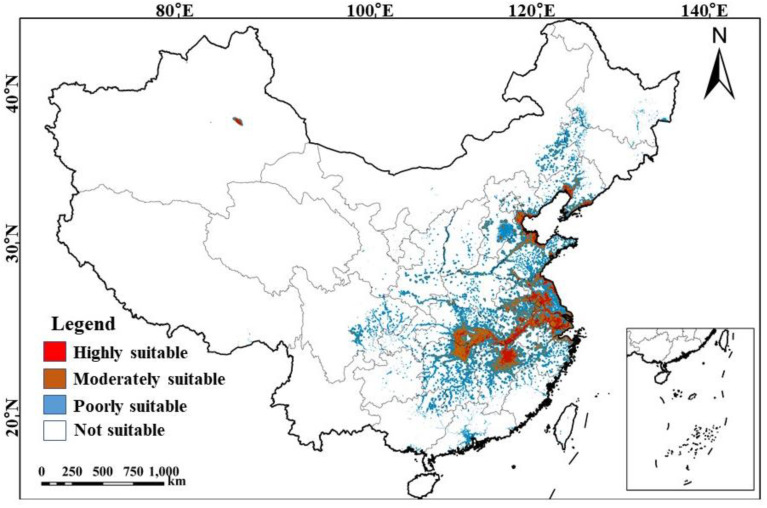
Potential distribution of suitable wintering areas for hooded cranes (note: highly suitable, moderately suitable, and poorly suitable represent areas where the habitat suitability index (HSI) is high (0.50 < *HSI* ≤ 1), moderate (0.20 < *HSI* ≤ 0.50), and poor (0.05 < *HSI* ≤ 0.20), respectively).

Currently, the suitable wintering areas for hooded cranes in China are primarily located in the middle and lower reaches of the Yangtze River, with additional suitable habitats found in parts of north and northeast China (Figure 4). The highly suitable area covers a total of 12.74 × 10^4^ km^2^, predominantly concentrated around inland lakes, such as Dongting Lake in Hunan Province, Liangzi Lake and Honghu Lake in Hubei Province, Poyang Lake in Jiangxi Province, Caizi Lake and Shengjin Lake in Anhui Province, Taihu Lake and Hongze Lake in Jiangsu Province. Coastal wetlands, including Chongming Dongtan in Shanghai, Panjin Wetland in Liaoning, Nandagang Wetland in Hebei, Qirihai Wetland in Tianjin, and the Yellow River Delta in Shandong, also contribute significantly to this area. The moderate-suitability area encompasses 27.35 × 10^4^ km^2^, primarily surrounding the high-suitability zones, with additional areas distributed in inland provinces, including the Xinjiang Uygur Autonomous Region and Henan Province. The poor-suitability area, totaling 83.06 × 10^4^ km^2^, is largely distributed around the moderate-suitability zones, with smaller distributions in some provinces of southwest, northeast, and south China.

### 3.5. Suitable Wintering Habitats for Hooded Cranes Under Future Climate and Land Use Scenarios

**Figure 5:**
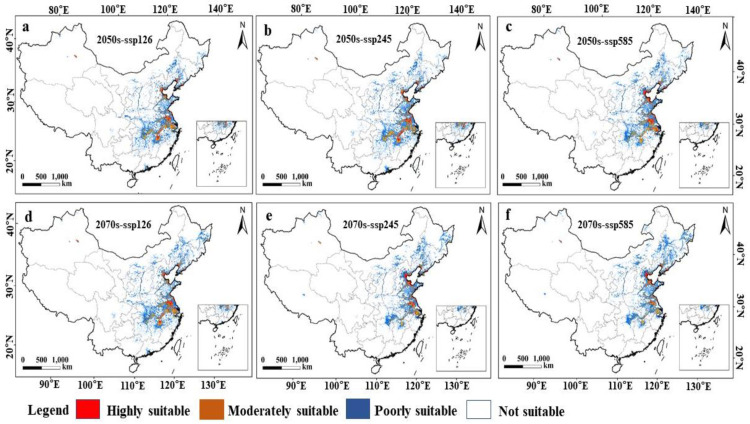
Distribution of suitable areas for hooded cranes under climate and land use scenarios in the future: (**a**) 2050s-ssp126; (**b**) 2050s-ssp245; (**c**) 2050s-ssp585; (**d**) 2070s-ssp126; (**e**) 2070s-ssp245; and (**f**) 2070s-ssp585 (note: highly suitable, moderately suitable, and poorly suitable represent areas where the habitat suitability index (HSI) is high (0.50 < *HSI* ≤ 1), moderate (0.20 < *HSI* ≤ 0.50), and poor (0.05 < *HSI* ≤ 0.20), respectively).

The distribution range of suitable areas (high, moderate, and poor suitability zones) for hooded cranes under future climate scenarios generally aligns with the current distribution, but overall, the suitable areas show significant contraction with minor expansion. The contracted area is between 3.49 and 12.27 times larger than the expanded area for each period (Appendix A Table A1, Figure 5). Across the SSP126, SSP254, and SSP585 scenarios, the average proportion of contracted areas is 29.1%, 28.8%, and 31.6%, respectively. Over time, the expansion areas slightly increase for SSP126, decrease for SSP585, and show substantial increases for SSP245. Meanwhile, the contraction areas remained nearly constant for SSP126 but increased significantly for SSP254 and SSP585. The highly suitable areas decrease notably across all three climate scenarios, with the largest reduction under SSP585 (2070s) and the smallest reduction under SSP585 (2050s) (Figure 5c and Figure 5f, respectively), reducing to 45.70% and 61.35% of the current area, respectively. The moderately suitable areas see only minor reductions across all three future climate scenarios. The low-suitability areas show a wide range of changes, with SSP126 (2070s) and SSP585 (2070s) experiencing the largest increases (Appendix A Table A1).

**Figure 6:**
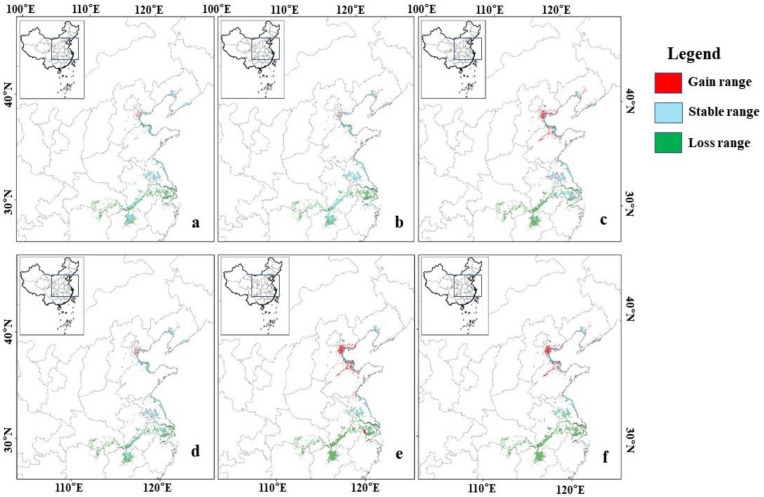
Changes in the distribution patterns of suitable habitats for hooded cranes under climate and land use change scenarios in the future: (**a**) 2050s-ssp126; (**b**) 2050s-ssp245; (**c**) 2050s-ssp585; (**d**) 2070s-ssp126; (**e**) 2070s-ssp245; and (**f**) 2070s-ssp585.

Spatially, the unchanged suitable habitats (high- and moderate-suitability areas) are mainly concentrated in Jiangxi, Zhejiang, Jiangsu, Liaoning, Shandong, Tianjin, and Hebei provinces. The contracted areas are primarily in Hunan, Hubei, Jiangxi, Anhui, and Jiangsu provinces, while the expansion areas are mainly in Shandong, Hebei, Tianjin, and Liaoning provinces (Figure 6). The land use change patterns indicate substantial contraction of natural habitats, such as arable land, forest, and water bodies, in future suitable habitats in the middle and lower reaches of the Yangtze River, alongside significant urban expansion. In the northern suitable habitats, including Shandong, Hebei, Tianjin, and Liaoning, arable land initially contracts but later expands, with notable town area expansion and slight increases in the water area (Appendix A Table A2).

**Figure A2:**
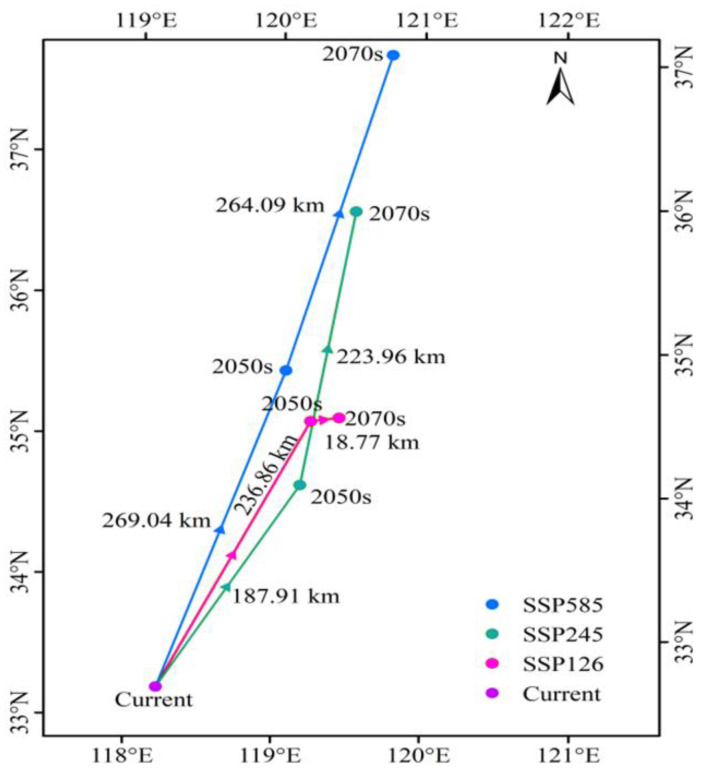
Centroid migration of suitable habitats for hooded cranes under climate and land use change scenarios in the future.

The centroid for suitable wintering habitats for hooded cranes shifts to the northeast under the future climate scenarios (Appendix A Figure A2). In the 2050s, it moves northeast under SSP126, SSP245, and SSP585, with the movement distances ranging from 187.91 to 269.04 km, in that order. In the 2070s, the centroid for SSP585 and SSP245 continues to shift northeastward by over 220 km, while SSP126 shifts only slightly eastward by approximately 18.77 km.

### 3.6. Changes in Temperature and Precipitation in the Future High Suitability Zone

The minimum temperature of the coldest month in the high-suitability zones for the wintering distribution of hooded cranes is projected to exhibit a gradual and steady increase under all three climate models as emissions intensify and time progresses. This warming trend is most pronounced in SSP585 (2070s), with a maximum temperature increase of 5.73 °C (Appendix A Table A3). Additionally, precipitation during the wettest month is anticipated to rise across all the future climate models (Appendix A Table A3).

## 4. Discussion

This study is the first to reveal the potential wintering distribution of the hooded crane in China, under both current and future conditions. The results show that, under future climate scenarios, the suitable habitat range will undergo significant changes, including a reduction in the habitat area of the hooded crane and a general shift toward the northeast.

### 4.1. Model Accuracy

The AUC value (0.977) of the present model reached an excellent level, indicating that the model can effectively predict the current and future wintering distribution pattern of hooded cranes [20]. The suitable areas for hooded cranes predicted by this model were also highly consistent with the current distribution sites. To ensure the high stability and accuracy of the model predictions, ArcGIS software was used to establish a 500 m buffer zone around the distribution location of hooded cranes to guarantee that only one site was present in each grid. In this study, the environmental variables were also tested for correlation, and the two variables with a correlation less than 0.8 were retained as the most influential environmental variables to ensure that the model predictions achieved a high level of precision.

### 4.2. Dominant Environmental Factors Affecting the Wintering Distribution of Hooded Cranes

The results of this study showed that the altitude, water environment (distance to major water, precipitation of the wettest month), and temperature (minimum temperature of the coldest month) were the dominant factors affecting the wintering distribution of hooded cranes. Altitude is an important influencing factor in terms of the distribution of many bird species [26,59]. High-altitude areas are characterized by cold temperatures and low oxygen levels, and they also lack developed water systems and large wetland habitats, limiting the distribution of most waterbirds [20]. Hooded cranes have a strong dependence on wetlands and prefer to overwinter in the low-altitude river and lake wetlands of the middle and lower Yangtze River [20,45]. In contrast, the black-necked crane (*Grus nigricollis*) prefers high-altitude wetlands, which is related to the differences in their diet and ecological habitats [20,45,60].

The results of this study also indicated that hooded cranes are highly dependent on water sources. The probability of overwintering presence decreases rapidly with increasing distance from major water bodies. The farther the distance from a water source, the less food hooded cranes can obtain. The precipitation during the wettest month has a significant effect on water levels and food resources in the wintering habitats of waterbirds. In south China, the wettest months typically occur in summer, when excessive rainfall raises water levels. These elevated water levels not only limit the growth of submerged plants but may also persist into winter, reducing food availability for hooded cranes and increasing the difficulty of foraging [26]. Conversely, insufficient precipitation in summer results in lower water levels in winter, exposing large areas of mudflats and causing the die-off of submerged plants [45]. Therefore, adequate precipitation during summer can provide suitable foraging and roosting environments for hooded cranes in winter.

The minimum temperature range of the coldest month suitable for hooded cranes to overwinter was −5.3 to 2.4 °C, which is much lower than that of Siberian cranes (*Leucogeranus leucogeranus*) in the same area (the minimum temperature range suitable for overwintering was 4.0 to 7.8 °C), indicating that hooded cranes are more adaptable to low temperatures [20,45]. Low temperatures can lead to ice formation in the water of the foraging habitat of hooded cranes, increasing the foraging difficulty, and insufficient food resources make it difficult for hooded cranes to obtain enough energy to cope with higher basal metabolism in winter [20,45,61].

### 4.3. Current Suitable Wintering Areas of Hooded Cranes

The primary wintering areas for hooded cranes in China are mainly located in in-land lakes in the middle and lower reaches of the Yangtze River, with a smaller number of high wintering areas found in the coastal wetlands of the North China Plain, the Shandong Peninsula, Bohai Bay, and the Liaodong Peninsula. The middle and lower reaches of the Yangtze River have a subtropical monsoon climate, which supports the growth of abundant aquatic vegetation in summer [46]. During the dry winter season, a large number of mudflats and shallow lakes are exposed, providing highly utilized food resources for wintering hooded cranes [45]. The high-suitability areas for hooded cranes in northern China are all located in the coastal wetlands of Shandong, Hebei, Tianjin, and Liaoning. This may be related to the high specific heat capacity of sea water in these coastal regions, where the freezing point is lower than that of freshwater. The high-suitability zone in the north has a temperature range of −17.5 to 3.4 °C. In the north, hooded cranes primarily forage in coastal wetlands and surrounding farmland, with their diet including wheat, corn, and plant roots. Some coastal wetlands remain unfrozen, and scattered crops in the surrounding farmland provide additional food resources for wintering hooded cranes. Influenced by the minimum temperature of the coldest month, the extremely low temperatures and extensive frozen water bodies in northeastern China may limit the current northward expansion of hooded cranes [62].

### 4.4. Suitable Wintering Areas of Hooded Cranes Under Future Climate and Land Use Change Scenarios

Temperature and precipitation are crucial factors influencing species distribution [61]. The results of this study indicate that the minimum temperature of the coldest month (−5.3 to 2.4 °C) and precipitation during the wettest month (178.2 to 328.5 mm) are the primary factors influencing the overwintering distribution of hooded cranes. The wintering distribution area of hooded cranes under future climate scenarios is largely consistent with the current distribution. The contracted areas are 3.49 to 12.27 times larger than the expanded areas in the corresponding periods, indicating that suitable habitats for hooded cranes will decrease in the future, with the contraction becoming more pronounced under the SSP245 (2070s) and SSP585 (2050s, 2070s) scenarios. The contracted areas are primarily located in the middle and lower reaches of the Yangtze River (Hunan, Hubei, Jiangxi, Anhui, Jiangsu, Shanghai), where temperatures gradually increase over time, leading to a significant decline in suitable habitats for hooded cranes. As the intensity of greenhouse gas emissions increases over time, the minimum temperature of the coldest month rises (Appendix A Table A3). Future temperature increases are a significant driver of changes in the wintering distribution of waterfowl [63]. A warmer climate will affect the distribution of aquatic plants and small aquatic animals (e.g., fish, crustaceans, and insects), which are the main food resources for waterbirds, and lead to the shifting of wintering sites [64]. Rising temperatures may also increase evaporation from many lakes in the middle and lower reaches of the Yangtze River during the dry season, intensifying the frequency of extreme droughts. This would lead to a continuous decline in the quality and area of natural habitats, thereby heightening the ecological risks for hooded cranes wintering in this region of China [26,65].

Habitat quality has a significant impact on the survival rate of wintering birds, and the loss or fragmentation of high-quality habitats increases the risk of bird extinction [26,66,67]. The high-suitability areas in the middle and lower reaches of the Yangtze River currently support most of the hooded crane population in China [36]. However, the future contraction of high-suitability areas in these regions is significant, greatly reducing the carrying capacity for the hooded cranes population. As the minimum temperature rises in the future, hooded cranes will move northward to habitats such as coastal wetlands, reservoirs, and surrounding farmlands at low elevations in China’s middle and high latitudes. The expansion areas are mainly located in the Shandong Peninsula, Bohai Bay coast, and Liaodong Peninsula, with a tendency for the coastal areas to extend into inland croplands (Figure 6). In the 2050s and 2070s, the mean minimum temperatures of the coldest month in the Shandong Peninsula, Bohai Bay coast, and Liaodong Peninsula are projected to be −5.08 °C (−12.27 to 1.33 °C) and −4.11 °C (−11.1 to 2.97 °C), which is similar to the current suitable minimum temperature range. The increase in temperature, along with the slight increase in water area, will make these areas more suitable for hooded cranes. With the availability of some food resources in the surrounding farmland, the energy intake of hooded cranes will be largely satisfied. Due to the easy accessibility of farmland food resources, more hooded cranes may be attracted to gather in the northern China. Continuous attention is needed as the situation evolves.

During the wintering period, hooded cranes dynamically adjusted their foraging habitat utilization patterns in response to the spatial and temporal variations in food resource availability. For instance, they preferentially utilized food-rich farmland habitats in the pre-wintering period to replenish energy, then shifted to grassland habitats during the mid- to late-winter period to store energy in preparation for migration, influenced by grain consumption and spring plowing activities [26,36]. An even mix of various habitat types is essential to meet the wintering needs of hooded cranes. However, in the northern regions, where wetlands are frozen, hooded cranes primarily feed on crops such as maize, rice, wheat, and soybeans in agricultural fields. As a result, any change in the distribution area of hooded cranes could dramatically alter their food composition. Despite the expansion of suitable areas in the north, these areas are much smaller and more fragmented than the contraction of traditional wintering grounds. Consequently, the survival of hooded cranes may face significant challenges in the future. However, whether the future expansion areas can support the wintering population of hooded cranes remains uncertain. This situation is similar to the case in the Mediterranean region, where natural habitats have been replaced by artificial habitats, yet large waterbird populations have still been maintained [68]. Further research is needed to explore this issue. Under the three climate models, the high wintering suitability areas for hooded cranes failed to expand northward to the Liaodong Peninsula. This limitation is primarily attributed to the extremely low temperatures in the northern regions of the Liaodong Peninsula. The small increase in rainfall and water areas in the north under the future climate emission models is also a significant factor contributing to the increase in suitability in this region (Appendix A Table A3). Therefore, this study suggests that the steady increase in the minimum temperature of the coldest month, the decrease in habitat quality in the traditional wintering areas, and the slight increase in the area of northern water bodies are the main factors influencing the large contraction and small expansion of the suitable wintering areas for hooded cranes.

Global warming has caused many species to migrate to higher altitudes and latitudes [20,69,70], with most waterbirds being constrained by their wetland distribution and typically migrating only toward higher latitudes [20]. Siberian cranes also exhibit northeasterly migration in response to climate warming, primarily driven by increases in the mean winter temperatures [20]. The distribution centroids for Siberian cranes in the 2050s and 2070s are projected to migrate northward by 77–178 km and 104–257 km, respectively. In contrast, the wintering area of the common cranes (*Grus grus*) population in Xinjiang is expected to migrate northward by approximately 2400 km compared to the traditional wintering area [20]. The findings of this study indicate that the centroid for hooded cranes in the 2050s and 2070s will shift northward by 187–269 km and 18–264 km, respectively (Appendix A Figure A2). This migration pattern is similar to that of Siberian cranes and differs significantly from that of common cranes [20], likely due to the stronger adaptive capacity of common cranes. In terms of the population size, diet, and distribution area, the ecological niche of the common crane is much broader than that of hooded cranes and Siberian cranes [71]. These results suggest that the migration of cranes to higher latitudes in response to temperature changes has become a common phenomenon, and the habitat conditions encountered by these species after migrating northward require careful attention. While the migration of hooded cranes brings their wintering areas closer to their breeding areas, the distance of the migration is limited. Compared to the energy savings and risks mitigated during migration, the significant reduction in suitable habitat area and the alterations in food composition following northward migration present more severe threats to the survival of hooded crane populations.

### 4.5. Limitations of the Study

While this study provides valuable predictions of the hooded crane distribution, certain limitations remain. For instance, the uneven distribution of citizen science data may impact the accuracy of species distribution models. However, we supplemented the citizen science data with reliable sources, mitigating the impact of data bias on the results. Although climate change is a key driver, other factors, such as interspecies interactions, migration capabilities, and food availability, also play significant roles but were not fully considered in this study [55,72]. This study assumes a fixed response to environmental changes without accounting for species’ evolutionary potential and adaptability, which could influence distribution patterns under climate change. Moreover, despite incorporating multiple climate variables, spatial and temporal data limitations may affect the model’s accuracy, especially due to insufficient or biased data in certain regions. To improve the accuracy and reliability of species distribution predictions, future research should integrate additional ecological factors, including interspecies interactions and evolutionary mechanisms, and utilize more comprehensive long-term datasets.

### 4.6. Conservation Recommendations

The impacts of climate change and human activities on avian habitats are profound, making the implementation of targeted conservation measures essential [11,15,59,73,74,75]. Based on the observed impacts on the wintering distribution of hooded cranes and projected future distribution trends, the following recommendations are made. (1) Strengthen hydrological monitoring and regulation: The distance to major water and precipitation during the wettest month are key factors influencing the distribution of hooded cranes. Therefore, enhancing water level regulation is crucial for the protection of the species. Water levels significantly impact the foraging behavior of hooded cranes, and extreme droughts or flooding can cause abnormal water levels in suitable habitats. Strengthening hydrological monitoring and maintaining optimal water levels will help create favorable conditions for foraging habitat. (2) Restoration of natural habitats: The quality of natural habitats for hooded cranes has been declining, leading to a shift toward artificial habitats, which increases the foraging risks. Restoring vegetation and improving the quality of natural habitats will provide sufficient food resources and optimize the structure of wintering habitats for hooded cranes. (3) Establishment of small nature reserves: As the wintering area of hooded cranes is projected to expand into regions such as Shandong Province, Tianjin City, and Liaoning Province, new nature reserves should be established to fill protection gaps in these regions and ensure adequate protection for the species. (4) Enhancement of the population monitoring and their suitable habitats: Under future climate scenarios, the area of suitable habitats, such as Poyang Lake and Shengjin Lake, is expected to shrink significantly. It is essential to enhance dynamic monitoring of hooded crane populations and their suitable habitats in these areas. This monitoring will inform the development of effective protection policies. Additionally, the quality and size of suitable habitats and the ecological habits of hooded crane populations in the northern expansion areas should be evaluated, with particular attention paid to food resources and diet monitoring.

## 5. Conclusions

Based on the MaxEnt model, the following conclusions were drawn. (1) The elevation, distance to major water sources, minimum temperature of the coldest month, and precipitation of the wettest month are the dominant environmental factors influencing the wintering distribution of hooded cranes in China. (2) The current high-suitability areas for the wintering distribution of hooded cranes in China are primarily located in the middle and lower reaches of the Yangtze River, the Shandong Peninsula, the Liaodong Peninsula, and other coastal areas. (3) The main wintering distribution areas of hooded cranes under future climate and land use change scenarios are similar to the current ones. However, in the future, the suitable habitats for hooded cranes will shrink significantly in the middle and lower reaches of the Yangtze River, while expanding slightly in the north. The centroid of the wintering distribution is expected to shift northeastward. (4) The temperature, habitat quality, and water body area play important roles in the current and future wintering distribution of hooded cranes. To better protect the hooded crane population and its habitat, future efforts should focus on restoring natural habitats and improving the management of artificial habitats to reduce the impact of human activities on the foraging and resting behaviors of hooded cranes.

## Figures and Tables

**Table 1:** Environmental variables and their descriptions.

Variable	Description	Unit
Bio1	Annual mean temperature	°C
Bio2	Mean diurnal temperature range	°C
Bio3	Isothermality	-
Bio4	Temperature seasonality	°C
Bio5	Max temperature of the warmest month	°C
Bio6	Min temperature of the coldest month	°C
Bio7	Temperature annual range	°C
Bio8	Mean temperature of the wettest quarter	°C
Bio9	Mean temperature of the driest quarter	°C
Bio10	Mean temperature of the warmest quarter	°C
Bio11	Mean temperature of the coldest quarter	°C
Bio12	Annual precipitation	mm
Bio13	Precipitation of the wettest month	mm
Bio14	Precipitation of the driest month	mm
Bio15	Precipitation seasonality (coefficient of variation)	mm
Bio16	Precipitation of the wettest quarter	mm
Bio17	Precipitation of the driest quarter	mm
Bio18	Precipitation of the warmest quarter	mm
Bio19	Precipitation of the coldest quarter	mm
Elev	Elevation	m
LUCC	Land use classification	-
DP	Distance to paddy field	m
DW	Distance to major water	m
DB	Distance to beach	m
DR	Distance to road	m
DV	Distance to village	m
Asp	Aspect	-
Slo	Slope	°

(note: Bio1–Bio19 and elevation data were obtained from WorldClim. LUCC data were obtained from Figshare. DP, DW, DB, DR, and DV were derived through ArcGIS Euclidean distance analysis. Asp and Slo was derived from ArcGIS slope analysis).

**Table 2:** Percentage contribution and permutation importance of the environmental variables affecting the wintering distribution of hooded cranes.

Environmental Variable	Percent Contribution (%)	Permutation Importance (%)
Elevation	43.7	26.6
Distance to major water	39.5	24
Min temperature of the coldest month	9.7	29.7
Precipitation of the wettest month	2.6	13.1
Slope	1.8	1.7
Isothermality	1.7	3.9
Land use classification	0.9	0.9

## Data Availability

The data presented in this study are available upon request from the corresponding author. The data are not publicly available due to the constraint in the consent.

## Appendix A

**Table A1:** Changes in the area and centroid of suitable wintering areas for hooded cranes under climate change scenarios.

Climatic Model	Phase	Area (km^2^)		Migration Distance (km)	High (×10^4^ km^2^)	Moderate (×10^4^ km^2^)	Poor (×10^4^ km^2^)
		Expansion	Contraction				
	Current				12.74	27.36	83.06
ssp126	2050s	5431.59	66,633.23	236.86	6.62	21.18	64.59
ssp126	2070s	8600.78	66,189.58	18.77	6.98	22.08	162.70
ssp245	2050s	8211.57	61,981.10	187.91	7.36	23.62	72.22
ssp245	2070s	21,323.78	78,757.51	223.96	7.00	19.14	14.25
ssp585	2050s	19,738.16	68,968.53	269.04	7.82	22.27	69.45
ssp585	2070s	17,679.10	86,811.95	264.09	5.82	18.98	73.74

**Table A2:** Future land use change of suitable habitats under climate change scenarios for hooded cranes.

Phase		Land Types					
		Cultivated Land (km^2^)	Forestland (km^2^)	Grassland (km^2^)	Towns (km^2^)	Bare Land (km^2^)	Water (km^2^)
Southern Suitable Habitats	Current	141,780.55	12,582.48	5592.44	17,450.54	212.81	28,647.7
	2050ssp126	70,957.29	1610.41	4757.56	19,594.01	231.23	22,745.25
	2070ssp126	65,343.36	1439.55	4691.06	29,344.46	218.95	22,047.48
	2050ssp245	83,585.81	2323.53	4679.81	19,567.4	237.37	21,877.64
	2070ssp245	38,004.25	438.92	3606.54	19,068.12	207.69	18,726.39
	2050ssp585	64,297.72	695.73	4178.47	21,457.13	219.97	20,603.84
	2070ssp585	26,335.42	321.26	4407.65	17,512.95	159.61	17,580.48
Northern suitable habitats	Current	34,040.63	2873.98	8664.91	7273.45	667.08	7744.09
	2050ssp126	25,377.77	3600.4	5731.59	16,127.63	756.09	7973.27
	2070ssp126	22,408.64	5341.77	6477.45	19,960.29	509.52	9289.02
	2050ssp245	33,207.8	3712.95	7214.11	15,239.55	822.59	9179.54
	2070ssp245	36,752.96	4975.49	8441.86	22,670.56	970.95	10,023.63
	2050ssp585	37,768.93	4740.17	7860.73	21,342.54	1137.72	9262.42
	2070ssp585	31,943.21	5224.11	8860.33	29,886.72	991.42	11,055.97

Note: south refers to wetlands in the middle and lower reaches of the Yangtze River; north refers to Shandong Province and areas north of it.

**Table A3:** Future changes in the minimum temperature of the coldest month and precipitation of the wettest month under climate and land use change scenarios.

Phase	Climate Model	Average Temperature (°C, with Range)	Average Precipitation (mm, with Range)
Current		−2.08 (−17.3–4.1)	198.79 (3.49–336)
2050s	ssp126	1.02 (−13.2–7.2)	215.53 (3–370)
	ssp245	1.38 (−12.4–7.6)	232.92 (3–362)
	ssp585	2.14 (−10.6–8.4)	225.74 (3–339)
2070s	ssp126	1.11 (−11–7)	217.01 (3–360.5)
	ssp245	2.62 (−10.5–8.7)	234.56 (3–377.5)
	ssp585	3.65 (−8.4–9.8)	244.37 (3–354)
